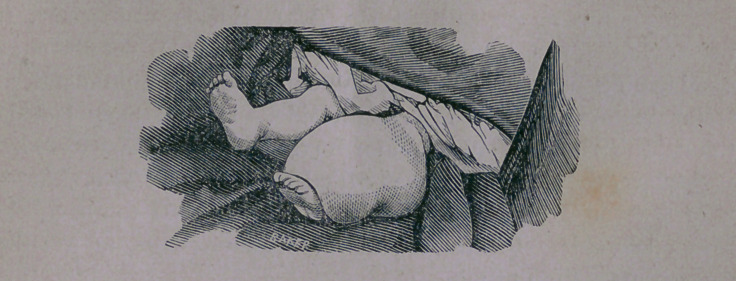# Congenital Fibro-Cellular Tumor

**Published:** 1869-12

**Authors:** Moses Gunn

**Affiliations:** Chicago, Ill.


ARTICLE III. — *Congenital Fibro-cellular Tumor.* By MOSES GUNN, M.D., Chicago, Ill.

THE above wood cut is from a photograph, and represents correctly the appearance of the tumor. The little patient was fifteen months old and of healthy appearance, but was unable to raise the, to him, ponderous leg. At birth, a tumor surrounded the leg completely, about midway between the knee and ankle joints. This is the testimony of the mother and an aunt of the child.

At the age of three months Prof. Freer saw the case, and describes the tumor as being about half its present size. Now the disease extends from above the knee to the foot, which it overlaps nearly to the toes, and measures, at its largest part, twenty and one-half inches. The skin is smooth, attenuated and pliable. The mass is elastic without fluctuation; but the elasticity varies at different points, being in some places firm and hard. Exploration gave vent to serum, which continued to flow for an hour. A small trocar yielded the same discharge, but no more freely than did the needle.

*Diagnosis.* — Hypertrophy of areolar tissue, with secretory endowment of the walls of the areolar spaces.

The disease was extending rapidly, and amputation was decided upon, and performed on the 6th of March last. Ether was administered, and the operation hardly produced a perceptible shock. The wound healed by granulation, mainly.

A section of the tumor showed the disease to be confined to the superficial fascia, the deep fascia and integument being normal.

The microscopic character of the tissue conformed to Paget's description of fibro-cellular growths.

## Figures and Tables

**Figure f1:**